# Survival after combined resection and ablation is not inferior to that after resection alone, in patients with four or more colorectal liver metastases

**DOI:** 10.1007/s00423-023-03082-1

**Published:** 2023-08-29

**Authors:** Iakovos Amygdalos, Lea Hitpass, Felix Schmidt, Gerrit Josephs, Jan Bednarsch, Marie-Luise Berres, Tom Lüdde, Steven W. M. Olde Damink, Tom Florian Ulmer, Ulf P. Neumann, Philipp Bruners, Sven Arke Lang

**Affiliations:** 1 Department of General, Visceral and Transplantation Surgery, University Hospital RWTH Aachen, Pauwelsstraße 30, 52074 Aachen, Germany; 2 Center for Integrated Oncology Aachen, Bonn, Cologne and Düsseldorf (CIO ABCD), Bonn, Germany; 3 Department of Diagnostic and Interventional Radiology, University Hospital RWTH Aachen, Aachen, Germany; 4 Department of Internal Medicine III, University Hospital RWTH Aachen, Aachen, Germany; 5 Department of Gastroenterology, Hepatology and Infectious Diseases, University Hospital Düsseldorf, Düsseldorf, Germany; 6 Department of Surgery, Maastricht University Medical Center, Maastricht, The Netherlands

**Keywords:** Colorectal liver metastases, Ablation, Survival, Hepatobiliary, Surgery

## Abstract

**Purpose:**

Colorectal liver metastases (CRLM) are the predominant factor limiting survival in patients with colorectal cancer. Multimodal treatment strategies are frequently necessary to achieve total tumor elimination. This study examines the efficacy of liver resection combined with local ablative therapy in comparison to liver resection only, in the treatment of patients with ≥ 4 CRLM.

**Methods:**

This retrospective cohort study was conducted at the University Hospital RWTH Aachen, Germany. Patients with ≥ 4 CRLM in preoperative imaging, who underwent curative resection between 2010–2021, were included. Recurrent resections and deaths in the early postoperative phase were excluded. Ablation modalities included radiofrequency or microwave ablation, and irreversible electroporation. Differences in overall- (OS) and recurrence-free-survival (RFS) between patients undergoing combined resection-ablation vs. resection only, were examined.

**Results:**

Of 178 included patients, 46 (27%) underwent combined resection-ablation and 132 (73%) resection only. Apart from increased rates of adjuvant chemotherapy in the first group (44% vs. 25%, *p* = 0.014), there were no differences in perioperative systemic therapy. Kaplan–Meier and log-rank test analyses showed no statistically significant differences in median OS (36 months for both, *p* = 0.638) or RFS (9 months for combined resection-ablation vs. 8 months, *p* = 0.921). Cox regression analysis showed a hazard ratio of 0.891 (*p* = 0.642) for OS and 0.981 (*p* = 0.924) for RFS, for patients undergoing resection only.

**Conclusion:**

For patients with ≥ 4 CRLM, combined resection-ablation is a viable option in terms of OS and RFS. Therefore, combined resection-ablation should be considered for complete tumor clearance, in patients with multifocal disease.

**Supplementary Information:**

The online version contains supplementary material available at 10.1007/s00423-023-03082-1.

## Introduction

Colorectal cancer (CRC) remains among the commonest and deadliest diseases worldwide, with up to 80% of CRC patients developing colorectal liver metastases (CRLM) [1, 2]. Curative liver resection ensures the best survival outcomes, but patients frequently present with complex disease, requiring multimodal treatment strategies [3]. These include multi-stage hepatectomies (MSH), portal vein embolization (PVE) or Associating Liver Partition and Portal vein ligation for Staged hepatectomy (ALPPS), local ablative or chemoembolization therapies and perioperative chemotherapy [4].

Local ablative therapy (LAT) plays an increasingly important role in the management of CRLM, as an adjunct to resection or standalone treatment [5, 6]. Common modalities include radiofrequency ablation (RFA), microwave ablation (MWA) and irreversible electroporation (IRE) [5–7], and can be conducted intraoperatively or percutaneously [3, 6–8]. Technological advancements and accumulated experience have resulted in ever-improving results [7, 8], so that LAT is now included in official guidelines for multimodal therapy of CRLM [3, 9]. However, evidence on the efficacy of LAT compared to resection remains limited and heterogeneous [6, 9], partly because studies have focused on LAT of irresectable lesions, with inherent bias regarding long-term outcomes [6, 10].

Data regarding combined resection and LAT for multiple CRLM is also sparse [11], despite the advantages of LAT being particularly pertinent in these patients, which were historically deemed unresectable [12–17]. Even now, multiple CRLM are considered a predictor of poor survival or increased recurrence risk [12–17]. Although attitudes shifted to consider these patients as surgically treatable, especially in combination with LAT [18], it has been shown that patients with ≥ 4 CRLM remain at a particular disadvantage [15, 19, 20].

The aim of this study was to compare overall- (OS) and recurrence-free (RFS) survival between patients undergoing combined resection-ablation or resection only, for ≥ 4 CRLM.

## Materials and methods

This study was conducted under ethical approval of the Institutional Review Board of the RWTH Aachen University (EK-001/21) and in accordance with the current version of the Declaration of Helsinki, the Declaration of Istanbul, and good clinical practice guidelines (ICHGCP). Informed consent was waived due to the retrospective study design and collection of readily available clinical data. Furthermore, the study was performed in accordance with the Strengthening the Reporting of Observational Studies in Epidemiology (STROBE) guidelines [21].

### Patient cohort and inclusion criteria

Consecutive adult patients with ≥ 4 CRLM undergoing elective liver resections in curative intent at the University Hospital RWTH Aachen between 2010 and 2021 were eligible for inclusion in this retrospective study. Patients operated for recurrences, those dying within 90 days postoperatively and those with unresected primary tumor (PT) or liver metastases were excluded. Patients were divided into those who underwent resection only (RES group), or additional intraoperative or percutaneous LAT (RESABL group). The primary and secondary endpoints were OS and RFS, respectively.

### Data collection

Data was obtained from a prospectively-maintained retrospective database [4]. The Union for International Cancer Control (UICC) tumor / lymph node /metastasis (TNM) system was used for PT staging and the Brisbane classification [22] was used to describe liver resections, which were designated major, when involving ≥ 3 segments. Preoperative computerized tomography (CT), magnetic resonance imaging (MRI) and positron emission tomography (PET) scans were used to determine the number, size, and location of CRLM, as well as the presence of extrahepatic metastases.

Metastases diagnosed within 3 months of the PT were defined as synchronous. Chemotherapy regimens were defined as previously described [4]: neoadjuvant before resection of rectum carcinoma; adjuvant after resection of advanced PT; inductive for initially unresectable CRLM; additive after liver resection, in patients with remaining systemic tumor load. Postoperative complications were stratified according to the Clavien-Dindo (CD) classification [23] and the Comprehensive Complication Index (CCI) [24].

### Operative technique

Operative technique followed common clinical standards [4]. Intraoperative ultrasonography confirmed preoperative imaging findings and excluded new manifestations. In open surgery, the Cavitron Ultrasonic Surgical Aspirator (CUSA®, Integra LifeSciences, Plainsboro NJ, USA) was used for parenchymal transection, with clipping or ligation of vascular and biliary structures. In laparoscopic cases, either the THUNDERBEAT (Olympus K.K., Tokyo, Japan), HARMONIC ACE® (Ethicon Inc. Somerville, NJ, USA) or laparoscopic CUSA® (Integra LifeSciences, Plainsboro NJ, USA) devices were employed, combined with ECHELON™ vascular staplers (Ethicon, Somerville, New Jersey, USA) or Weck® Hem-o-lok® polymer clips (Teleflex Inc., Pennsylvania, USA). Pringle maneuvers were applied as needed. Anatomical or parenchyma-sparing resections were chosen according to general patient condition, preoperative liver function tests, and limiting factors, such as macrovascular invasion. Resection margins were controlled intraoperatively with frozen section examination.

### Ablative technique

Ablations were performed intraoperatively under ultrasonography guidance, or percutaneously under CT control, in general anesthesia. Manufacturer recommendations and standardized treatment protocols were strictly adhered to. For RFA, a monopolar system (RF 3000, Boston Scientific, Marlborough, MA, USA) was used, with a 480 kHz frequency and variable output up to 200W through an umbrella-shaped electrode (LeVeen, Boston Scientific, Marlborough, MA, USA). Depending on lesion size, an array diameter of 2 cm-5 cm was used to ensure complete lesion coverage, including a safety margin of ≥ 5 mm. Ablation procedures comprised two cycles, separated by a 120-s pause. Each cycle stopped when tissue impedance reached > 500Ω. For MWA, the Emprint Ablation System with Thermosphere Technology (Medtronic, Dublin, Ireland) was used. Ablation parameters and antenna shaft length (15 cm or 20 cm) were chosen according to size and location of the target lesion, aiming for a 5 mm safety margin. Finally, IRE was conducted using unipolar, 19-gauge probes (NanoKnife, AngioDynamics, Latham, New York, USA) with an active tip length of 15 mm-25 mm. The number of probes depended on target lesion size and intended margin width. Under electrocardiographic gating, 70 pulses of 90 µs duration were applied per probe pair, with 3000 V maximum voltage. To confirm successful ablation and exclude complications, a triphasic liver CT was performed after each LAT.

### Indication for ablation

Treatment strategies were set in multidisciplinary team meetings. For technically irresectable lesions, LAT was generally combined with systemic therapy and resection of further hepatic metastases, if present. Frequently, IRE was employed to protect vessels and bile ducts near target lesions. For resectable lesions, LAT was utilized to avoid staged resections in patients with comorbidities or to minimize loss of liver parenchyma, where disproportionately large resections were required. Concepts were often individualized, based on patient and tumor characteristics. The decision to ablate was sometimes made intraoperatively, for example, when new, functionally irresectable lesions were identified.

### Propensity-score-matching analysis

To address our study’s inherent selection bias, a propensity score matching (PSM) analysis was carried out. Variables chosen for matching were age, sex, ASA score, synchronicity of metastases, chemotherapy regimens, major and multi-stage resections, number of metastases and MDLL.

### Statistical analysis

Groups were compared using Mann–Whitney U, Chi-square, or Fisher’s exact tests. Independent risk factors for OS and RFS were identified through uni- and multivariable Cox regression analyses and hazard ratios (HR) were given with 95% confidence intervals (CI). Factors with *p* < 0.10 in the univariable analysis were considered for inclusion in the multivariable model. Median follow-up time was calculated using the reverse Kaplan–Meier technique. Differences in survival were compared with Kaplan–Meier analysis and the log-rank test. Results were reported as medians and interquartile range (IQR, given as 1^st^-3^rd^ quartiles) for continuous variables, or absolute and relative frequencies for categorical and ordinal variables. All *p*-values < 0.05 were considered statistically significant. Statistical analysis and graph generation was performed using SPSS Statistics v29 (IBM Corp., Armonk, NY, USA) and Prism v9.0 (GraphPad Software, La Jolla, CA, USA), respectively.

## Results

### Patient characteristics

**Fig. 1:**
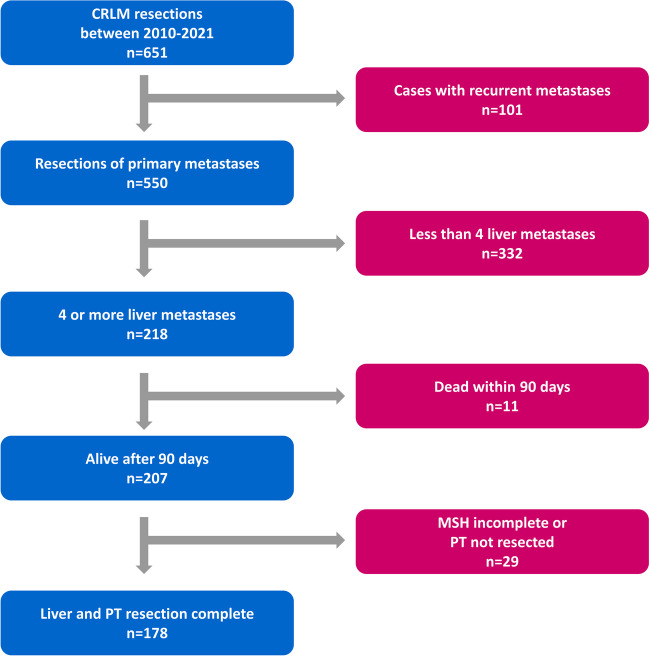
Flowchart of inclusion and exclusion criteria, leading to the final study population. Abbreviations: CRLM, colorectal liver metastases; MSH, multi-stage hepatectomy; PT, primary tumor

One hundred seventy-eight patients were included (Fig. 1), with a median age of 62 (53–67) years. The commonest PT sites were rectum (46%), sigmoid (24%) and ascending colon (14%). Most PT were staged T3 (68%) and/or N1 (40%). Metastases were overwhelmingly bilateral (87%) and mostly synchronous (79%). The RESABL group comprised 46 (26%) patients. The RES group had more synchronous metastases (83% vs. 67%, *p* = 0.030), fewer lesions (67% vs 48% with 4–6 lesions, 17% vs 39% with 7–9 lesions, *p* = 0.007), larger median diameter of the largest lesion (MDLL, 3.0 cm vs. 2.2 cm, *p* = 0.01), higher preoperative serum carcinoembryonic antigen (CEA, 7.0 µg/l vs. 4.0 µg/l, *p* = 0.03) and lower rates of adjuvant chemotherapy (25% vs. 44%, *p* = 0.01), Detailed information is provided in Table 1. Fifty-three ablative procedures were carried out, of which 21(40%) were RFA, 20 (37%) MWA and 12(23%) IRE. Six (13%) patients underwent > 1 LAT, including a simultaneous left-sided IRE and right-sided RFA for one patient (Table 2).

**Table 1:** Demographic and oncological information of whole cohort, RESABL and RES groups

	All patients (*n* = 178)	RESABL (*n* = 46)	RES (*n* = 132)	*p-*value
Age	62 (53–67)	62 (53–67)	61 (52–67)	0.92
Sex (M)	110 (62%)	33 (72%)	77 (58%)	0.11
BMI	25.0 (23.0–28.0)	26.0 (24.0–29.0)	25.0 (22.0–28.0)	0.14
ASA score				0.35
I	5 (3%)	1 (2%)	4 (3%)	
II	69 (39%)	13 (28%)	56 (42%)	
III	96 (54%)	30 (65%)	66 (50%)	
IV	8 (4%)	2 (5%)	6 (5%)	
Primary tumor location				0.96
Coecum	14 (8%)	5 (11%)	9 (7%)	
Ascending colon	25 (14%)	7 (15%)	18 (14%)	
Transverse colon	4 (2%)	1 (2%)	3 (2%)	
Descending colon	10 (6%)	2 (4%)	8 (6%)	
Sigmoid colon	42 (24%)	11 (24%)	31 (23%)	
Rectum	83 (46%)	20 (44%)	63 (48%)	
Primary tumor diff. grade				0.12
G1	3 (2%)	1 (3%)	2 (2%)	
G2	120 (83%)	29 (94%)	91 (80%)	
G3	21 (15%)	1 (3%)	20 (18%)	
Primary tumor T-stage				0.30
T1	5 (3%)	3 (7%)	2 (2%)	
T2	17 (10%)	5 (11%)	12 (9%)	
T3	119 (68%)	30 (67%)	89 (68%)	
T4	34 (19%)	7(15%)	27 (21%)	
Primary tumor *N*-stage				0.72
N0	43 (25%)	13 (30%)	30 (23%)	
N1	68 (40%)	16 (36%)	52 (41%)	
N2	61 (35%)	15 (34%)	46 (36%)	
Synchronous metastases	140 (79%)	31 (67%)	109 (83%)	0.03
Number of metastases				0.007
4–6	111 (62%)	22 (48%)	89 (67%)	
7–9	40 (23%)	18 (39%)	22 (17%)	
≥ 10	27 (15%)	6 (13%)	21 (16%)	
Location of metastases				0.13
Only right (Seg. V-VIII)	22 (12%)	2 (4%)	20 (15%)	
Only left (Seg I-IV)	1 (1%)	0 (0%)	1 (1%)	
Bilateral	155 (87%)	44 (96%)	111 (84%)	
Diameter of largest metastasis (cm)	2.7 (1.8–4.1)	2.2 (1.6–3.1)	3.0 (1.8–4.7)	0.01
Extrahepatic metastases				0.91
None	143 (80%)	37 (80%)	106 (80%)	
Pulmonary	17 (10%)	4 (9%)	13 (10%)	
Skeletal	1 (1%)	0 (0%)	1 (1%)	
Other (incl. peritoneal)	15 (8%)	4 (9%)	11 (8%)	
Combined	2 (1%)	1 (2%)	1 (1%)	
*KRAS* status (mutated)	54 (40%)	16 (46%)	38 (38%)	0.45
Preoperative serum CEA (µg/l)	6.3 (3.0–30.8)	4.0 (2.9–7.5)	7.0 (3.0–38.0)	0.03
Perioperative chemotherapy (yes)^a^				
Neoadjuvant	38 (22%)	11 (24%)	27 (21%)	0.59
Adjuvant	53 (30%)	20 (44%)	33 (25%)	0.01
Inductive	103 (58%)	24 (52%)	79 (60%)	0.36
Additive	51 (29%)	10 (22%)	41 (31%)	0.25
Liver R0	154 (87%)	40 (87%)	113 (86%)	0.91
Primary tumor R0	159 (97%)	41 (98%)	118 (97%)	0.77

Legend: Values given as median (1st quartile – 3rd quartile) or absolute and relative frequencies; Abbreviations used: *RESABL* Resection combined with ablation; *RES* Resection only; *BMI* Body Mass Index; *ASA* American Society of Anesthesiology score; *KRAS Kirsten rat sarcoma viral oncogene homolog*; *CEA* Carcinoembryonic antigen; ^a^Chemotherapy regimens defined as: neoadjuvant before rectum resection, adjuvant after colon resection, inductive before liver resection, additive after liver resection. Overlapping percentages due to patients undergoing multiple regimens

**Table 2:** Summary of ablative procedures

Modality	*n*	LR	*p*-value
Number of patients undergoing ablations:	46		
• only intraoperative	7 (15%)		
• only percutaneous	37 (81%)		
• both	2 (4%)		
Number of ablation procedures:	53	25 (47%)	0.88^b^
• RFA intraoperative	2 (4%)	1 (50%)	
• RFA percutaneous^a^	19 (36%)	8 (42%)	
• MWA intraoperative	7 (12%)	4 (57%)	
• MWA percutaneous	13 (25%)	6 (46%)	
• IRE intraoperative	2 (4%)	1 (50%)	
• IRE percutaneous^a^	10 (19%)	5 (50%)	
Number of patients with 2 ablations	4 (9%)		
Number of patients with 3 ablations	2 (4%)		

Legend: Values given as median (1st quartile – 3rd quartile) or absolute and relative frequencies; Abbreviations used: *LR* Local recurrence; *RFA* Radiofrequency ablation; *MWA* Microwave ablation; *IRE* Irreversible electroporation. ^a^One patient underwent combined RFA of a right-sided lesion, and IRE of a left-sided lesion; ^b^Local recurrence rate compared between RFA / MWA / IRE, combining intraoperative and interventional ablations

### Operative data and postoperative morbidity

Intra- and perioperative data is summarized in Table 3. Sixty-eight per cent of patients underwent major resections and 26% required MSH, including ALPPS. Patients in the RES group underwent significantly more major resections (75% vs. 48%, *p* < 0.001), especially extended hepatectomies or trisectionectomies (24% vs. 0%, *p* < 0.001). On the other hand, patients in the RESABL group underwent up to 3 atypical resections significantly more often (26% vs 7%, *p* < 0.001). The RES group underwent significantly more anatomical resections (38% vs. 15%, *p* = 0.005), whereas the opposite was true for atypical resections (37% vs. 15%, *p* = 0.002). Finally, there was a significantly higher proportion of patients without complications (CD = 0) in the RESABL group (24% vs. 11%, *p* = 0.038), as well as a significantly lower median CCI (15.0 vs. 24.2, *p* = 0.019).

**Table 3:** Operative data and postoperative morbidity of whole cohort, RESABL and RES groups

	All patients (*n* = 178)	RESABL (*n* = 46)	RES (*n* = 132)	*p-*value
Portal vein embolization	50 (28%)	9 (20%)	41 (31%)	0.14
Major resections	121 (68%)	22 (48%)	99 (75%)	< 0.001
Operation details				
≤ 3 atypical resections	21 (12%)	12 (26%)	9 (7%)	< 0.001
> 3 atypical resections	16 (9%)	5 (11%)	11 (8%)	0.61
Bisegmentectomy	17 (9%)	5 (11%)	12 (9%)	0.72
Hemihepatectomy	44 (25%)	11 (24%)	33 (25%)	0.88
Ext. hemihep. / Trisectionectomy	31 (17%)	0 (0%)	31 (24%)	< 0.001
Staged resection	33 (19%)	11 (24%)	22 (17%)	0.28
ALPPS	13 (7%)	1 (2%)	12 (9%)	0.12
Other	3 (2%)	1 (2%)	2 (1%)	0.77
Operation technique				
Minimally invasive	27 (15%)	8 (17%)	19 (14%)	0.63
Converted	7 (4%)	1 (2%)	6 (4%)	0.48
Open	134 (75%)	33 (72%)	101 (77%)	0.52
Combined	10 (6%)	4 (9%)	6 (4%)	0.29
Resection type				
Anatomical	57 (32%)	7 (15%)	50 (38%)	0.005
Atypical	37 (21%)	17 (37%)	20 (15%)	0.002
Combined	84 (47%)	22 (48%)	62 (47%)	0.92
Operation time (min)	294 (224–361)	266 (205–348)	303 (227–368)	0.097
Intraoperative transfusions				
Red blood cells	0 (0–1)	0 (0–1)	0 (0–1)	0.90
Fresh frozen plasma	0 (0–3.3)	0 (0–4)	0 (0–3)	0.63
Platelets	0 (0–0)	0 (0–0)	0 (0–0)	0.30
Complications				
CD-0	26 (14%)	11 (24%)	15 (11%)	0.038
CD-I	44 (25%)	13 (28%)	31 (24%)	0.52
CD-II	46 (26%)	10 (22%)	36 (27%)	0.46
CD-IIIa	28 (16%)	4 (9%)	24 (18%)	0.13
CD-IIIb	18 (10%)	3 (6%)	15 (11%)	0.35
CD-IVa	14 (8%)	4 (9%)	10 (8%)	0.81
CD-IVb	2 (1%)	1 (2%)	1 (1%)	0.43
CCI	22.6 (8.7–37.4)	15.0 (8.7–27.6)	24.2 (12.2–38.2)	0.019

Legend: Values given as median (1st quartile – 3rd quartile) or absolute and relative frequencies; Abbreviations used: *RESABL*, resection combined with ablation; *RES*, resection only; *ALPPS*, Associating liver partition and portal vein ligation for staged hepatectomy; *CD*, Clavien-Dindo grade; *CCI*, comprehensive complication index

### Survival analysis

**Fig. 2:**
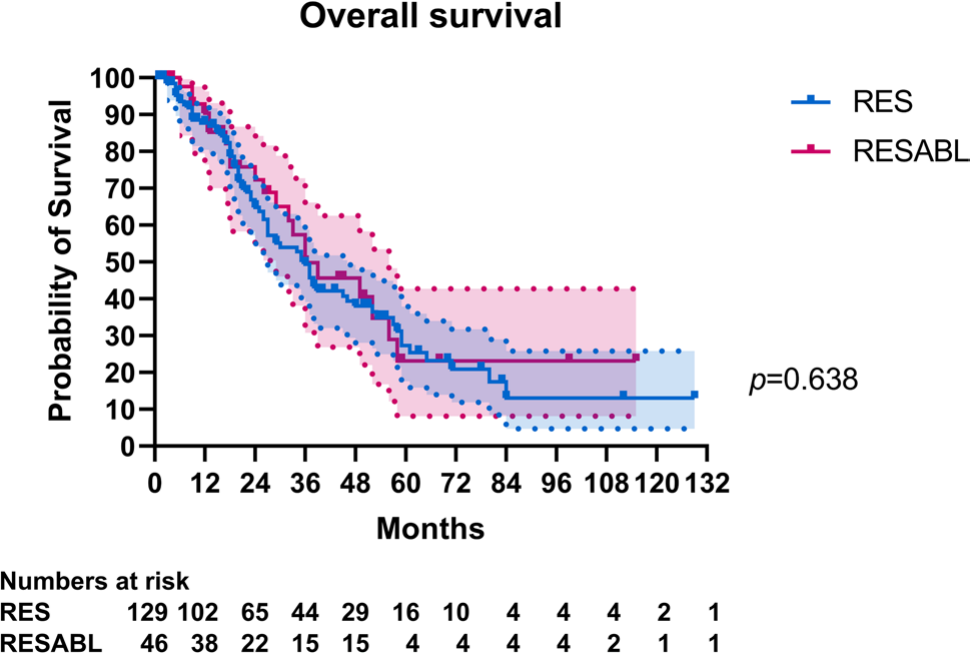
Comparison of overall survival between RESABL and RES groups

**Fig. 3:**
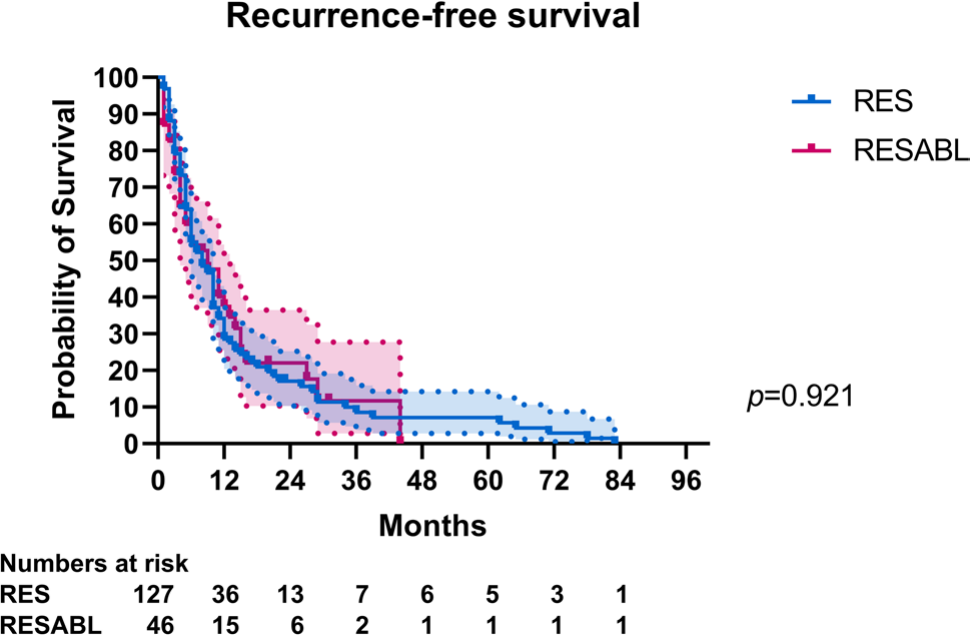
Comparison of recurrence-free survival between RESABL and RES groups

Median follow-up was 50 months, with no differences between the two groups (*p* = 0.28). Median OS and RFS for the study cohort were 36 months (95%CI 31–41) and 8 months (95%CI 6–10), respectively. There were no differences in median OS (RESABL: 36 months, 95%CI 19–53, vs. RES: 36 months, 95%CI 28–44, *p* = 0.64) or RFS (RESABL: 9 months, 95%CI 4–14, vs. RES: 8 months, 95%CI 6–10, *p* = 0.92) between the two groups (Figs. 2 and 3). Furthermore, there were no differences in median OS (RFA 52 months vs. MWA 39 months vs. IRE 32 months, *p* = 0.248) or RFS (RFA 14 months vs. MWA 6 months vs. IRE 4 months, *p* = 0.111) according to ablation type (Supplementary Fig. 1). Moreover, there were local recurrences after 25 (47%) ablative procedures, with the median local-recurrence-free survival (LRFS) being 19 months for all 53 procedures and no significant differences in local recurrence rates between ablation modalities (Table 2). As shown in Supplementary Fig. 2, there were no differences in median LRFS according to ablation type (RFA 20 months vs. MWA 18 months vs. IRE 20 months, *p* = 0.664).

As shown in Tables 4 and 5, inductive chemotherapy was an independent risk factor for OS (HR 1.761, 95%CI 1.006–3.066, *p* = 0.047) and RFS (HR 1.488, 95%CI 1.042–2.125, *p* = 0.03), and bilateral metastases for RFS only (HR 1.886, 95%CI 1.069–3.328, *p* = 0.03). Treatment strategy (RESABL or RES) was neither a risk factor for OS (*p* = 0.64), nor for RFS (*p* = 0.92).

**Table 4:** Cox regression—Association of demographic and oncological factors with overall survival

Perioperative factor	Univariable cox regression analysis		Multivariable cox regression analysis	
	HR (95% CI)	*p*-value	HR (95% CI)	*p*-value
Resection only	0.891 (0.548–1.449)	0.64		
Age	1.025 (1.004–1.047)	0.02	1.014 (0.987–1.041)	0.32
Sex (F)	0.802 (0.525–1.228)	0.31		
BMI	0.973 (0.929–1.019)	0.24		
ASA score	1.188 (0.858–1.645)	0.30		
Prim. tumor diff. grade	1.975 (1.080–3.613)	0.02	1.835 (0.944–3.567)	0.07
Prim. tumor T-stage	1.316 (0.943–1.836)	0.11		
Prim. tumor N-stage	0.931 (0.707–1.224)	0.61		
*KRAS* (mutated)	1.585 (1.015–2.476)	0.04	1.592 (0.947–2.677)	0.08
Preoperative serum CEA (µg/l)	1.000 (1.000–1.001)	0.17		
Multi-stage hepatectomy (yes)	1.151 (0.732–1.811)	0.54		
Number of metastases (increasing)	1.073 (1.012–1.137)	0.02	1.076 (0.973–1.191)	0.16
Location of metastases (bilateral)	1.492 (0.791–2.813)	0.22		
Diameter of largest metastasis (increasing)	1.095 (1.019–1.176)	0.01	1.085 (0.993–1.186)	0.07
Portal vein embolization	0.965 (0.617–1.509)	0.88		
Neoadjuvant chemotherapy^a^	0.859 (0.512–1.439)	0.56		
Adjuvant chemotherapy^a^	0.980 (0.620–1.552)	0.93		
Inductive chemotherapy^a^	1.561 (1.020–2.389)	0.04	1.761 (1.006–3.066)	0.047
Additive chemotherapy^a^	0.747 (0.479–1.163)	0.20		
Liver R0	0.739 (0.431–1.268)	0.27		
Primary tumor R +	2.751 (0.858–8.819)	0.09		
Extrahepatic metastases (yes)	1.446 (0.894–2.341)	0.13		
Primary tumor location	0.907 (0.800–1.028)	0.13		
Synchronous / metachronous (metachronous)	0.936 (0.571–1.535)	0.79		
Major / minor resections (minor)	0.902 (0.581–1.399)	0.65		

Legend: Results given as hazard ratios (HR) with 95% confidence intervals (95% CI). Factors with *p* < 0.10 in the univariable analysis were considered for inclusion in the multivariable Cox regression model. To avoid a multicollinearity effect, not all eligible variables were included in the multivariable logistic regression analysis; Abbreviations used: *BMI* Body Mass Index; *ASA* American Society of Anesthesiology score; *KRAS Kirsten rat sarcoma viral oncogene homolog*; *CEA* Carcinoembryonic antigen; ^a^Chemotherapy regimens defined as: neoadjuvant before rectum resection, adjuvant after colon resection, inductive before liver resection, additive after liver resection

**Table 5:** Cox regression—association of demographic and oncological factors with recurrence-free survival

Perioperative factor	Univariable cox regression analysis		Multivariable cox regression analysis	
	HR (95% CI)	*p*-value	HR (95% CI)	*p*-value
Resection only	0.981 (0.667–1.444)	0.92		
Age	0.998 (0.982–1.014)	0.79		
Female sex	1.093 (0.771–1.550)	0.62		
BMI	1.001 (0.961–1.042)	0.97		
ASA score	1.181 (0.897–1.554)	0.24		
Prim. tumor diff. grade	1.360 (0.852–2.171)	0.20		
Prim. tumor T-stage	1.241 (0.953–1.616)	0.11		
Prim. tumor N-stage	1.250 (0.993–1.572)	0.06	1.211 (0.965–1.520)	0.10
*KRAS* mutation	1.353 (0.925–1.979)	0.12		
Preoperative serum CEA (µg/l)	1.000(1.000–1.001)	0.66		
Multi-stage hepatectomy	1.223 (0.836–1.789)	0.30		
Number of metastases (increasing)	1.058 (1.001–1.117)	0.04	1.030 (0.973–1.089)	0.31
Bilateral metastases	2.027 (1.169–3.515)	0.01	1.886 (1.069–3.328)	0.03
Diameter of largest metastasis (increasing)	1.023 (0.956–1.094)	0.51		
Portal vein embolization	1.121 (0.777–1.617)	0.54		
Neoadjuvant chemotherapy^a^	0.698 (0.454–1.073)	0.10		
Adjuvant chemotherapy^a^	0.998 (0.691–1.444)	0.99		
Inductive chemotherapy^a^	1.603 (1.133–2.268)	0.008	1.488 (1.042–2.125)	0.03
Additive chemotherapy^a^	0.924 (0.642–1.328)	0.67		
Liver R0	1.007 (0.611–1.658)	0.98		
Primary tumor R +	0.916 (0.338–2.486)	0.86		
Extrahepatic metastases (yes)	1.463 (0.982–2.180)	0.06	1.355 (0.903–2.034)	0.14
Primary tumor location	1.010 (0.911–1.119)	0.85		
Metachronous disease	0.887 (0.598–1.316)	0.55		
Minor resections	1.176 (0.823–1.681)	0.37		

Legend: Results given as hazard ratios (HR) with 95% confidence intervals (95% CI). Factors with *p* < 0.10 in the univariable analysis were considered for inclusion in the multivariable Cox regression model. To avoid a multicollinearity effect, not all eligible variables were included in the multivariable logistic regression analysis; Abbreviations used: *BMI* Body Mass Index; *ASA* American Society of Anesthesiology score; *KRAS Kirsten rat sarcoma viral oncogene homolog*; *CEA* Carcinoembryonic antigen; ^a^Chemotherapy regimens defined as: neoadjuvant before rectum resection, adjuvant after colon resection, inductive before liver resection, additive after liver resection

A subgroup analysis was carried out on patients with bilateral CRLM (n = 155, 87%), to further investigate the effect on survival. Bilateral metastasis was associated with significantly reduced RFS (7 months vs. 12 months, *p* = 0.008), but not OS (36 months vs. 39 months, *p* = 0.21), compared to the rest of the cohort (Supplementary Fig. 3). However, in patients with bilateral metastases, no differences between the RESABL and RES groups regarding OS (36 months vs. 35 months, *p* = 0.78) or RFS (9 months vs. 6 months, *p* = 0.63) were observed (Supplementary Fig. 4).

### Propensity-score-matching analysis

The PSM analysis resulted in 44 matched pairs, leaving two patients in the RESABL group unmatched, with a total cohort size of 88. As can be seen in Supplementary Table 1, most previously significant differences between the groups have either no or reduced statistical significance after PSM. The only exception are major resections, which even after PSM are significantly increased in the RES group (*p* < 0.001). Survival analysis still showed no differences in median OS (RES 29 months vs. RESABL 39 months, *p* = 0.292) or RFS (RES 6 months vs. RESABL 9 months, *p* = 0.797), after PSM (Supplementary Fig. 5). Cox regression excluded the treatment group as a predictor of OS or RFS, with HR of 0.745 (95%CI 0.427–1.300, *p* = 0.294) and 0.941 (95%CI 0.582–1.524, *p* = 0.805), respectively.

## Discussion

In this study, we compared combined resection-ablation with resection alone, in patients with ≥ 4 CRLM, undergoing curative-intent treatment. We found no difference in OS or RFS between the groups, suggesting that multimodal therapy is a viable alternative to resection alone.

A higher rate of synchronous metastases, higher median CEA, larger MDLL, and higher rate of major resections were seen in the RES group. The higher rate of synchronous disease is probably linked to the treatment of PT and CRLM, which may involve multiple operations. This provides an opportunity to resect bilateral metastases in multiple steps, obviating the need for LAT. On the contrary, combining resection with LAT may spare multiple operations in patients with metachronous disease. The higher rate of synchronous disease would explain the higher median CEA, stemming from the combined tumor burden of PT and metastases. Furthermore, the larger MDLL reflects the limits of LAT regarding target lesion size. Generally, ablation of CRLM larger than 3 cm is not recommended, regardless of LAT modality [25]. Finally, the higher rate of major resections is probably due to large metastases, which were non-amenable to LAT or other parenchyma-sparing techniques.

In the RESABL group, more patients with ≥ 7 lesions and a higher rate of adjuvant chemotherapy were observed. As the chances of deep-lying or irresectable lesions are increased in patients with multiple lesions, adjunct LAT is more often applied. Finally, the higher rate of adjuvant therapy is probably linked to the frequency of metachronous metastases. In the case of resectable synchronous disease, there is no proven benefit of adjuvant therapy after resection of PT and CRLM (unless remaining systemic tumor or other risk factors are present) [3]. On the contrary, patients with UICC Stage III CRC and/or risk factors receive adjuvant treatment as standard, after which metachronous metastases may be diagnosed. Therefore, the group with more metachronous metastases should also exhibit higher rates of adjuvant therapy after resection of the PT.

So far, results from studies comparing resection and LAT for CRLM are inconsistent. Some reports show no difference in 3- or 5-year OS or RFS after propensity-score- [6, 26–28] or case-matching [29]. On the contrary, a systematic review by Kron et al. summarizing 18 non-randomized studies for resection vs. RFA, found a significantly better OS, RFS, and local recurrence rate in patients undergoing surgery [5]. However, these studies were heterogeneous regarding ablation modality (RFA, MWA or both) and route (laparoscopic, open, or interventional), were partly in the early 00’s (before modern chemotherapy and tumor biology concepts), and some investigated fairly small cohorts. Others compared MWA against combined resection-MWA, with contradicting results. For example, Stattner et al. demonstrated a significantly reduced OS and RFS with MWA alone [30], while Philips et al. found a longer median OS in the MWA group, and no difference in RFS [31]. None of these studies included resection alone [30, 31]. To date, few randomized controlled trials (RCT) have investigated this topic. The LAVA trial compared RFS after resection or ablation in high-risk surgical patients eligible for resection, but closed prematurely because of poor recruitment [32]. Final results are pending from the COLLISION RCT, which includes patients with at least one resectable and ablatable CRLM, ≤ 3 cm in diameter [33].

Contrary to our study, some previous reports have shown superior OS and RFS for patients with CRLM undergoing resection, compared to combined resection-ablation. Particularly, Abdalla et al. [34] and Gleisner et al. [35] investigated RFA alone, RFA combined with resection, and resection alone. Both studies showed significantly better OS and RFS for resection, compared to the other options [34, 35]. However, they did not focus on patients with multiple metastases and their cohorts stemmed mainly from the 90’s to early 00’s, before modern chemotherapy regimens were introduced. Similarly, Amerongen et al. found better 5-year OS and RFS in patients undergoing resection, compared to combined resection and intraoperative RFA [36]. However, they excluded patients undergoing MSH or synchronous resections of PT and CRLM, and those with extrahepatic disease. Additionally, significantly fewer R0 resections were reported in the combined group [36]. Other studies, including a meta-analysis by Meijerink et al., found no significant differences in OS or RFS between combined resection-ablation and resection alone [10, 37–39].

Regarding patients with ≥ 4 CRLM, De Jong et al. investigated a subgroup (*n* = 192) of an international cohort and demonstrated a higher risk of intrahepatic recurrence in those undergoing combined resection-ablation, compared to resection alone. However, no difference in OS or 5-year survival was observed [11]. More recently, Masuda et al. compared the two treatment strategies in a patient cohort divided into < 4 (*n* = 568) and ≥ 4 (*n* = 149) lesions [40]. Combined resection with RFA resulted in poorer OS in the < 4 lesions group, whereas no difference was seen in patients with ≥ 4 lesions. Additionally, *KRAS* mutation, positive PT N-status, and extrahepatic metastases were independent predictors of poor survival, contrary to our study. In agreement with our study, the combination of resection with RFA was not a predictor of poor prognosis [40]. A limitation of both studies is the inclusion of patients operated before modern chemotherapy regimens and concepts of tumor biology, especially in the first study, whose cohort originated between 1984–2009.

Our analysis precluded treatment strategy (RESABL or RES) as a risk factor for OS or RFS. In fact, only inductive chemotherapy was an independent risk factor fo r both measures of survival. One might speculate, that this was an indicator of advanced, initially irresectable disease, rather than a biological effect of the chemotherapy itself. The same association between inductive chemotherapy and OS was previously observed in our data [4]. Regarding RFS only, bilateral metastasis proved to be an additional independent risk factor. To further investigate this, we conducted a subgroup analysis in patients with bilateral disease, which found no differences in OS or RFS between the RESABL or RES groups. From this we conclude, that the increased recurrence risk in bilateral CRLM is an effect of tumor load and biology, rather than the choice of treatment strategy.

Some studies investigated the effect of risk factors, such as tumor biology, on survival after combined resection-ablation. For example, Sasaki et al. compared combined resection-ablation to resection alone and stratified patients according to risk factors, such as PT N-status, *KRAS* status and bilateral metastases [41]. Patients stratified as low-risk had similar 5-year OS to the resection-only group, whereas outcomes in the high-risk group were significantly worse [41]. In our study, *KRAS* status was only significant in the univariable analysis (trending towards significance in the multivariable analysis) for OS. Moreover, PT N-status was not a predictor of OS or RFS. As mentioned above, bilateral metastases were an independent risk factor for RFS. Furthermore, a study by Vles et al. demonstrated worse OS and local tumor progression rates in patients with non-desmoplastic histopathological growth patterns undergoing combined resection-ablation for CRLM [42]. We currently have no such information regarding our cohort.

Our study has certain strengths, such as its focus on patients with ≥ 4 metastases. This is particularly relevant in an era of increasingly aggressive multimodal strategies, for the treatment of patients with multiple CRLM. Compared to ours, many of the aforementioned studies are older, before modern chemotherapy regimens and surgical techniques became widespread, and some even excluded staged resections, such as ALPPS. We included all operative strategies and demonstrated homogeneity regarding the frequency of major resections, MSH, and PVE, between the two groups. Certain limitations should be considered when interpreting the results of this study, including its retrospective design, monocentric nature, and modest cohort size. Additionally, we analyzed different ablation modalities, conducted both intraoperatively and percutaneously, which may exhibit different outcomes when analyzed individually. However, this reflects modern clinical practice, where various LAT are used, according to tumor localization and general treatment concept. Additionally, there is an inherent bias in our study, as generally only irresectable tumors were ablated, or those that would significantly increase the extent of liver resection. Finally, as with any retrospective study, missing data is also a problem.

Despite these limitations, we have shown combined resection-ablation to be a viable solution for patients with multiple CRLM, which does not confer any disadvantages in terms of OS or RFS. In general, oncological results after RFA / MWA are continuously improving and the two modalities are similar in terms of local disease control [8]. More data is required concerning the role and efficacy of IRE in CRLM, which is promising, especially in cases where thermal ablation is contraindicated [7]. In any case, LAT presents a viable treatment option, particularly for metastases in unfavorable localizations, as its parenchyma-sparing properties allow for re-resection or re-ablation of recurrences [7, 43]. Further studies are warranted, especially prospective, multicentric RCT. Results from the aforementioned COLLISION trial are eagerly awaited. Meanwhile, well-designed retrospective or non-randomized prospective studies are necessary to further explore the role of LAT in patients with CRLM.

## Conclusion

The combination of resection with LAT constitutes a viable treatment option in patients with multiple (≥ 4) CRLM. Overall- and recurrence-free survival in these patients is comparable to those undergoing resection alone. Further studies are necessary to better define the role of LAT in the multimodal treatment of patients with CRLM.

### Supplementary Information

Below is the link to the electronic supplementary material.

Supplementary file1 (DOCX 30 kb)

Supplementary file2 (DOCX 240 kb)

## Data Availability

Data can be made available upon reasonable request to the corresponding author.

## Abbreviations

ALPPS: Associating liver partition and portal vein ligation for staged hepatectomy
ASA: American society of anesthesiology
BMI: Body mass index
CCI: Comprehensive complication index.
CD: Clavien-Dindo grade
CEA: Carcinoembryonic antigen
CI: Confidence interval
CRC: Colorectal cancer
CRLM: Colorectal liver metastases
CT: Computerized tomography
CUSA: Cavitron ultrasonic surgical aspirator
DFS: Disease-free survival
ESMO: European society for medical oncology
HR: Hazard ratio
ICU: Intensive care unit
IMC: Intermediate care unit
IQR: Interquartile range
IRE: Irreversible electroporation
KRAS: Kirsten rat sarcoma viral oncogene homolog
LAT: Local ablative therapy
LRFS: Local-recurrence-free survival
MDLL: Median diameter of the largest lesion
MRI: Magnetic resonance imaging
MSH: Multi-stage hepatectomy
MWA: Microwave ablation
OS: Overall survival
PET: Positron emission tomography
PT: Primary tumor
PVE: Portal vein embolization
PSM: Propensity score matching
R0: Resection margin negative
R1: Resection margin microscopically positive
RAS: Rat sarcoma viral oncogene homolog
RCT: Randomised controlled trial
RES: Resection-only group
RESABL: Combined resection-ablation group
RFA: Radiofrequency ablation
RFS: Recurrence-free survival
TNM: Tumor, lymph node, metastasis
UICC: Union for international cancer control
