# Supplementary material for: Survival after combined resection and ablation is not inferior to that after resection alone, in patients with four or more colorectal liver metastases
Source: Langenbecks Arch Surg. 2023 Aug 29;408(1):343. doi: 10.1007/s00423-023-03082-1 (PMC10465667; doi:10.1007/s00423-023-03082-1)
Supplement: Supplementary file 2 — Supplementary file2 (DOCX 240 kb) [file 423_2023_3082_MOESM2_ESM.docx]

**Supplement to:**

Survival after combined resection and ablation is not inferior to that after resection alone, in patients with four or more colorectal liver metastases.

Authors & Affiliations

Iakovos Amygdalos MBBS PhD ^1,2^, Lea Hitpass MD^2,3^, Felix Schmidt^1^, Gerrit Josephs^1^, Jan Bednarsch MD^1,2^, Marie-Luise Berres MD^2,4^, Tom Lüdde MD^2,5^, Steven W. M. Olde Damink MD PhD^1,6^, Tom Florian Ulmer MD^1,2^, Ulf P. Neumann MD^1,2,6^, Philipp Bruners MD^2,3^, and Sven Arke Lang MD^1,2^

^1^Department of General, Visceral and Transplantation Surgery, University Hospital RWTH Aachen, Aachen, Germany; ^2^Center for Integrated Oncology Aachen, Bonn, Cologne and Düsseldorf (CIO ABCD); ^3^Department of Diagnostic and Interventional Radiology, University Hospital RWTH Aachen, Aachen, Germany; ^4^Department of Internal Medicine III, University Hospital RWTH Aachen, Germany; ^5^Department of Gastroenterology, Hepatology and Infectious Diseases, University Hospital Düsseldorf, Germany; ^6^Department of Surgery, Maastricht University Medical Center, Maastricht, The Netherlands

Corresponding Author

Iakovos Amygdalos, MBBS BSc PhD

Department of General, Visceral and Transplantation Surgery;

University Hospital RWTH Aachen, Pauwelsstraße 30, 52074 Aachen, Germany

Tel: +49 (0) 241 80 37274; Fax: +49 (0) 241 80 82417

URL: [www.ukaachen.de](https://www.ukaachen.de/), e-mail: [iamygdalos@ukaachen.de](mailto:iamygdalos@ukaachen.de), Twitter: @iamygdalos

**Supplementary Table 1.** Demographic and oncological information of whole cohort, RESABL and RES groups, after propensity score matching.

|  | All patients  (n=88) | RESABL  (n=44) | RES  (n=44) | *p-*value |
| --- | --- | --- | --- | --- |
| Age | 62 (52-68) | 62 (53-68) | 62 (51-68) | 0.822 |
| Sex (M) | 58 (66%) | 31 (71%) | 27 (61%) | 0.368 |
| BMI | 26.0 (23.0-29.0) | 26.0 (24.0-29.0) | 25.0 (21.0-29.0) | 0.206 |
| ASA score  I  II  III  IV | 1 (1%)  35 (40%)  50 (57%)  2 (2%) | 1 (2%)  13 (30%)  28 (64%)  2 (5%) | 0 (0%)  22 (50%)  22 (50%)  0 (0%) | 0.110 |
| Primary tumor location  Coecum  Ascending Colon  Transverse Colon  Descending Colon  Sigmoid Colon  Rectum | 8 (9%)  16 (18%)  1 (1%)  4 (4%)  19 (22%)  40 (46%) | 4 (9%)  7 (16%)  1 (2%)  2 (5%)  11 (25%)  19 (43%) | 4 (9%)  9 (20%)  0 (0%)  2 (5%)  8 (18%)  21 (48%) | 0.873 |
| Primary tumor diff. grade  G1  G2  G3 | 1 (1%)  60 (90%)  6 (9%) | 1 (3%)  29 (94%)  1 (3%) | 0 (0%)  31 (86%)  5 (14%) | 0.185 |
| Primary tumor T-stage  T1  T2  T3  T4 | 3 (3%)  12 (14%)  55 (64%)  16 (19%) | 3 (7%)  5 (11%)  30 (68%)  6 (14%) | 0 (0%)  7 (17%)  25 (59%)  10 (24%) | 0.192 |
| Primary tumor N-stage  N0  N1  N2 | 21 (25%)  34 (41%)  28 (34%) | 13 (30%)  16 (37%)  14 (33%) | 8 (20%)  18 (45%)  14 (35%) | 0.548 |
| Synchronous metastases | 65 (74%) | 29 (66%) | 36 (82%) | 0.089 |
| Number of metastases  4-6  7-9  ≥10 | 52 (59%)  26 (30%)  10 (11%) | 20 (45%)  18 (41%)  6 (14%) | 32 (73%)  8 (18%)  4 (9%) | 0.030 |
| Location of metastases  Only right (Seg. V-VIII)  Only left (Seg I-IV)  Bilateral | 11 (12%)  0 (0%)  77 (88%) | 2 (4%)  0 (0%)  42 (96%) | 9 (20%)  0 (0%)  35 (80%) | 0.050 |
| Diameter of largest metastasis (cm) | 2.6 (1.6-3.8) | 2.2 (1.7-3.1) | 3.0 (1.6-4.6) | 0.076 |
| Extrahepatic metastases  None  Pulmonary  Skeletal  Other (incl. peritoneal)  Combined | 70 (80%)  9 (10%)  1 (1%)  7 (8%)  1 (1%) | 35 (80%)  4 (9%)  0 (0%)  4 (9%)  1 (2%) | 35 (80%)  5 (11%)  1 (2%)  3 (7%)  0 (0%) | 0.689 |
| *KRAS* status (mutated) | 33 (49%) | 16 (46%) | 17 (53%) | 0.544 |
| Preoperative serum CEA (µg/l) | 4.8 (2.6-30.4) | 4.1 (3.1-8.2) | 14.1 (1.9-99.0) | 0.163 |
| Perioperative chemotherapy (yes)^a^  Neoadjuvant  Adjuvant  Inductive  Additive | 21 (24%)  29 (33%)  47 (53%)  22 (25%) | 11 (25%)  20 (46%)  22 (50%)  9 (21%) | 10 (23%)  9 (21%)  25 (57%)  13 (30%) | 0.803  0.023  0.521  0.325 |
| Portal vein embolization | 25 (28%) | 9 (21%) | 16 (36%) | 0.098 |
| Major resections | 60 (68%) | 22 (50%) | 38 (86%) | <0.001 |
| Multi-stage hepatectomy | 25 (28%) | 12 (27%) | 13 (30%) | 0.813 |
| Liver R0 | 78 (89%) | 38 (86%) | 40 (91%) | 0.502 |
| Primary tumor R0 | 78 (95%) | 41 (98%) | 37 (93%) | 0.347 |

Legend: Values given as median (1st quartile – 3rd quartile) or absolute and relative frequencies; Abbreviations used: *RESABL*, resection combined with ablation; *RES*, resection only; *BMI*, Body Mass Index; *ASA*, American Society of Anesthesiology score; *KRAS*, *Kirsten rat sarcoma viral oncogene homolog*; *CEA*, Carcinoembryonic antigen; ^a^Chemotherapy regimens defined as: neoadjuvant before rectum resection, adjuvant after colon resection, inductive before liver resection, additive after liver resection. Overlapping percentages due to patients undergoing multiple regimens.

**Supplementary Figure 1.** OS and RFS according to ablation type


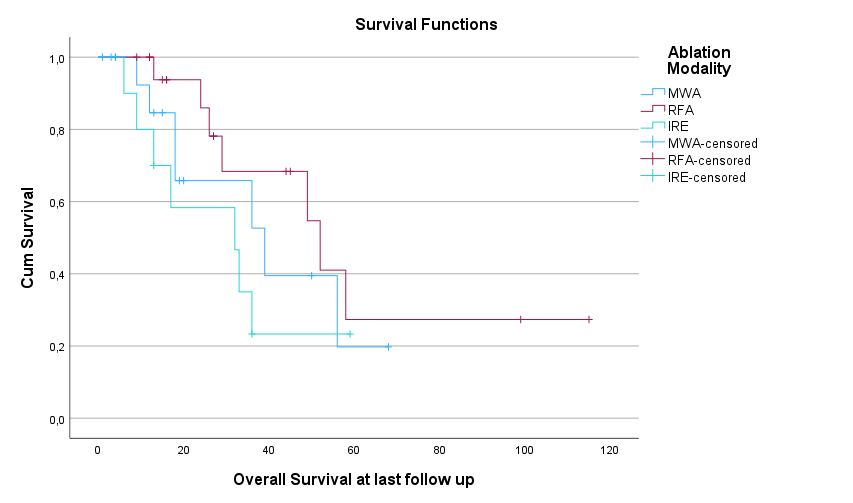


Median OS: RFA 52 months vs. MWA 39 months vs. IRE 32 months, *p*=0.248


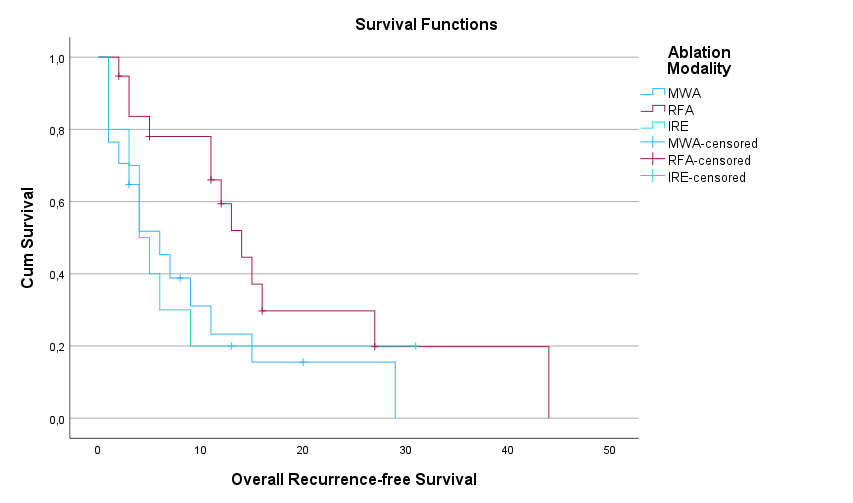


Median RFS: RFA 14 months vs. MWA 6 months vs. IRE 4 months, *p*=0.111

**Supplementary Figure 2.** Local-recurrence-free survival of whole cohort and according to ablation type

**
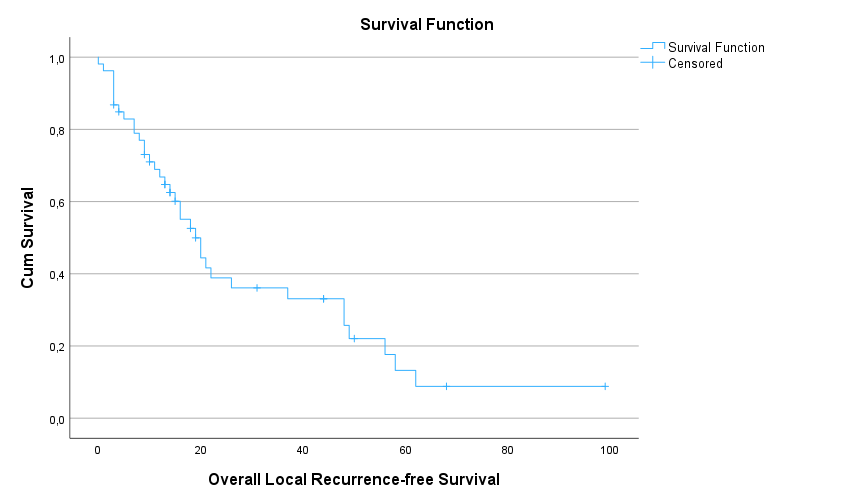
**

Median LRFS for whole cohort: 19 months

**
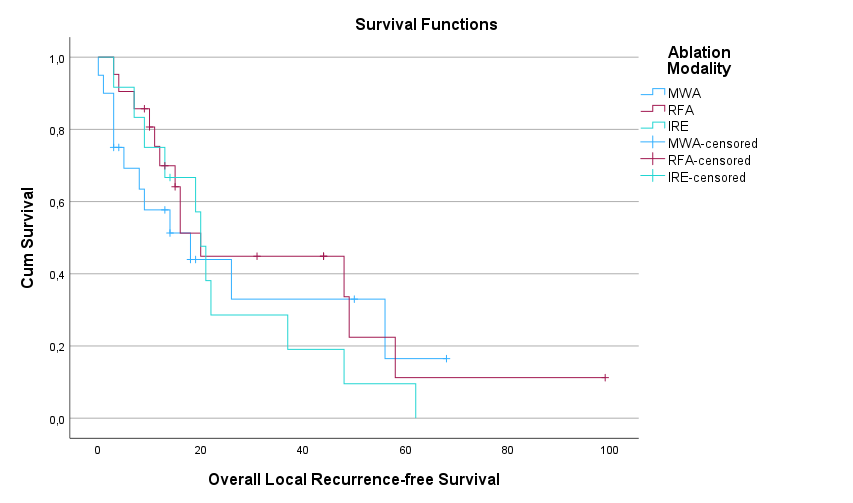
**

Median LRFS: RFA 20 months vs. MWA 18 months vs. IRE 20 months, *p*=0.664

**Supplementary Figure 3.** OS and RFS in one-sided vs. bilateral metastases

**
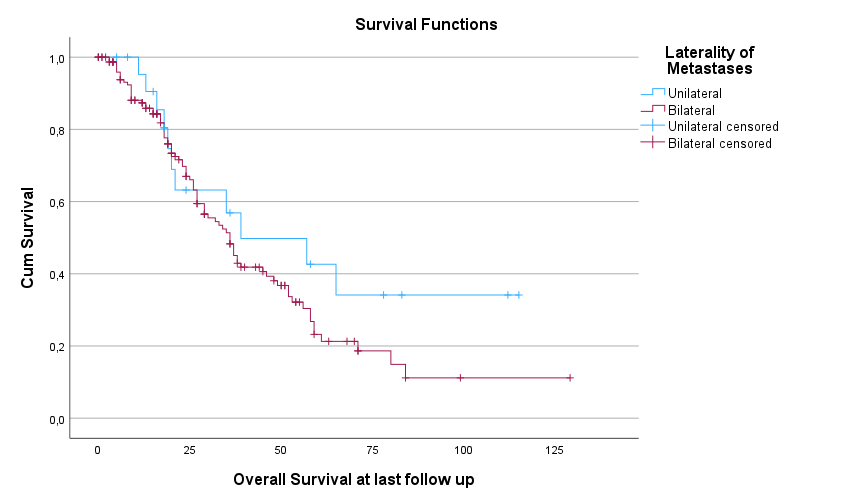
**

Median OS: One-sided 39 months vs. bilateral 36 months, *p*=0.209


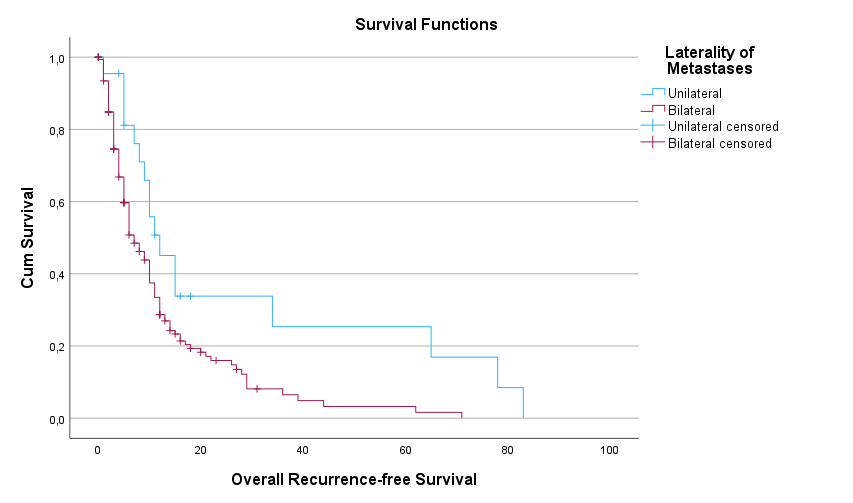


Median RFS: One-sided 12 months vs. bilateral 7 months, *p*=0.008

**Supplementary Figure 4.** OS and RFS of RESABL vs. RES in bilateral metastases only

**
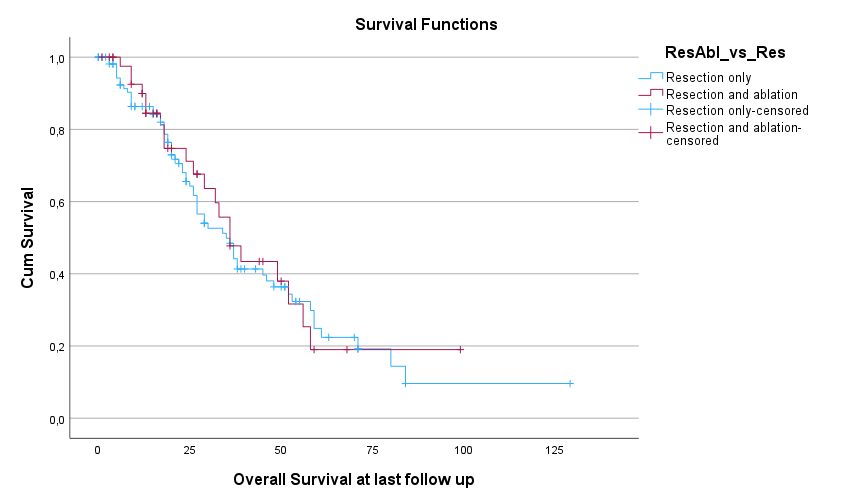
**

Median OS: RES 35 months vs. RESABL 36 months, *p*=0.780

**
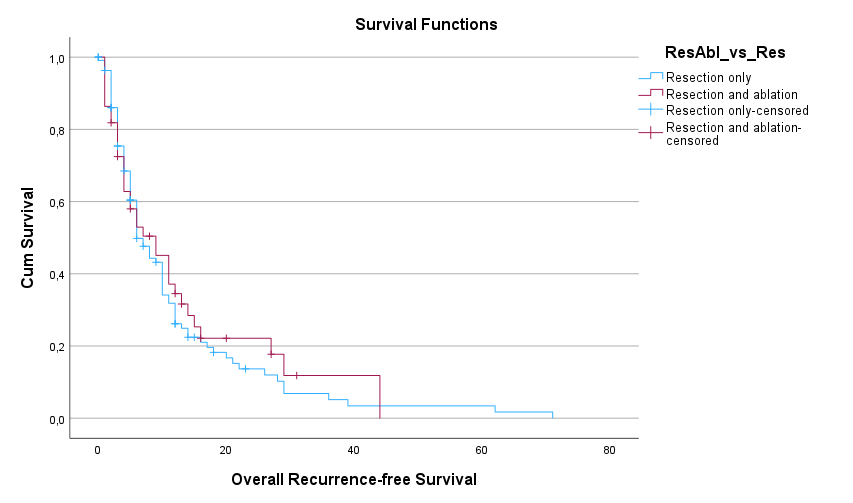
**

Median RFS: RES 6 months vs. RESABL 9 months, *p*=0.632

**Supplementary Figure 5.** OS and RFS of RESABL vs. RES after propensity score matching


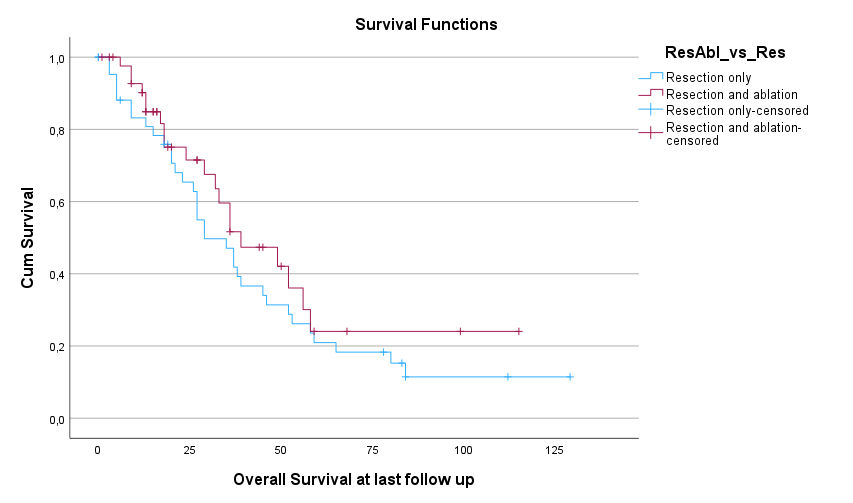


Median OS: RES 29 months vs. RESABL 39 months, *p*=0.292


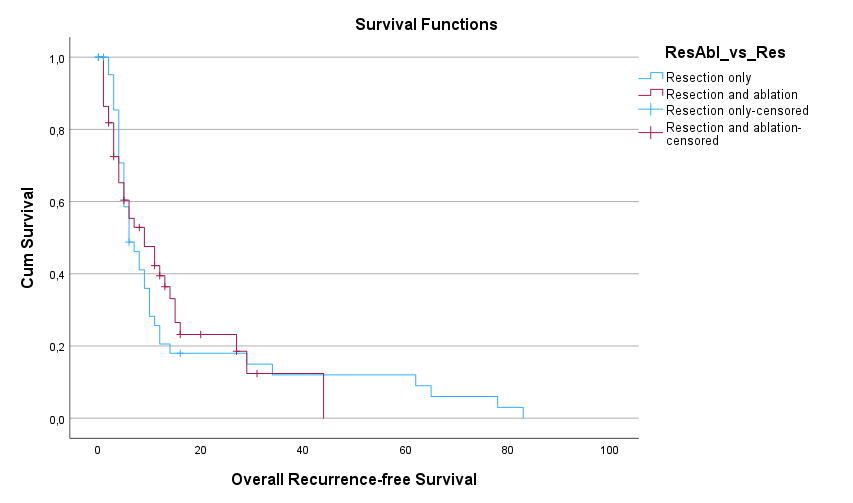


Median RFS: RES 6 months vs. RESABL 9 months, *p*=0.797
